# Complications to 6 months following total hip or knee arthroplasty: observations from an Australian clinical outcomes registry

**DOI:** 10.1186/s12891-020-03612-8

**Published:** 2020-09-10

**Authors:** Sung Mu Heo, Ian Harris, Justine Naylor, Adriane M. Lewin

**Affiliations:** 1 Hornsby-Kuringai Hospital, Palmerston Road, Hornsby, Sydney, NSW 2077 Australia; 2 Whitlam Orthopaedic Research Centre, Ingham Institute for Applied Medical Research, South Western Sydney Clinical School, UNSW, Sydney, Australia

**Keywords:** Total hip arthroplasty, Total knee arthroplasty, Primary joint replacement, Registry, Epidemiology, Complication, Readmission, Reoperation, Infection

## Abstract

**Background:**

Total hip and total knee arthroplasty (THA/TKA) are increasing in incidence annually. While these procedures are effective in improving pain and function, there is a risk of complications.

**Methods:**

Using data from an arthroplasty registry, we described complication rates including reasons for reoperation and readmission from the acute period to six months following THA and TKA in an Australian context. Data collection at 6 months was conducted via telephone interview, and included patient-reported complications such as joint stiffness, swelling and paraesthesia. We used logistic regression to identify risk factors for complications.

**Results:**

In the 8444 procedures included for analysis, major complications were reported by 9.5 and 14.4% of THA and TKA patients, respectively, whilst minor complications were reported by 34.0 and 46.6% of THA and TKA patients, respectively. Overall complications rates were 39.7 and 53.6% for THA and TKA patients, respectively. In THA patients, factors associated with increased risk for complications included increased BMI, previous THA and bilateral surgery, whereas in TKA patient factors were heart disease, neurological disease, and pre-operative back pain and arthritis in a separate joint. Female gender and previous TKA were identified as protective factors for minor complications in TKA patients.

**Conclusion:**

We found moderate rates of major and high rates of minor postoperative complications following THA and TKA in Australia and have identified several patient factors associated with these complications. Efforts should be focused on identifying patients with higher risk and optimising pre- and post-operative care to reduce the rates of these complications.

## Background

Total hip and total knee arthroplasty (THA, TKA) are effective interventions for osteoarthritis and are increasing in incidence annually. The Australian National Joint Replacement Registry reported 49,764 THA and 65,266 TKA procedures in 2018 [1]. Over 90,000 THA and over 1.1 million TKA procedures were undertaken in 2018 in the UK, whilst in the US, incidence is estimated to grow to 572,000 THA and 3.48 million TKA procedures per annum by 2030 [2]. While these procedures are effective in improving pain and function [3–7], there is a risk of complications. The most common complications requiring readmission for hip arthroplasty are dislocation and infection, whereas infection dominates following knee arthroplasty [8, 9]. Measuring and understanding rates of these events comprise important components of both informed consent and shared decision-making.

Complication rates following THA and TKA are reported using a broad range of metrics and time points. Reported complication rates vary widely, partly based on inclusion criteria (e.g. ‘major’ versus ‘minor’ complications), reporting base (e.g. single-centre cohorts versus nationwide registries), and length of follow up [10–14]. For example, 90-day venous thromboembolism rates reported in a nationwide database analysis in South Korea were 3.9% for THA and 3.8% for TKA, whereas in-hospital symptomatic DVT rates as reported in a systematic review were 0.26 and 0.63% for THA and TKA respectively, and PE rates at 0.14% and 0. 27% for THA and TKA respectively [15, 16]. Further, readmission rates following THA and TKA range from 0.27% from a single centre at 30 days post-operatively to 10.5 and 8.6% at 90 days for THA and TKA respectively in large analyses using US Medicare national hospital claims data [8, 9, 17].

Regarding overall complication rates, the American College of Surgeons National Surgery Quality Improvement Program (NSQIP) reported that at 30 days post-THA and TKA, 4.2 and 5.55% of people experience a major complication i.e. a complication requiring complex medical intervention, and minor complications occurred in 2.17 and 2.86%, respectively [6, 18]. In Australia, the Bureau of Health Information published readmission rates of 9 and 12% within 60 days of THA and TKA [19]. However, there are currently no published reports that capture the rates of complications following TKA or THA across multiple providers and include both the acute hospital stay along with post-discharge follow-up to 6 months post-surgery. Capturing and understanding complete rates of minor and major complications help to highlight potential failings in quality of care.

The aims of this study were to use data from an arthroplasty registry to describe complete complication rates from the acute period to six months following THA and TKA in an Australian context and to describe reasons for reoperation and readmission six months post-surgery. An additional aim was to identify risk factors for complications such as readmission and reoperation, following THA and TKA.

## Methods

### Study design and population

This retrospective cohort study included elective primary total hip and total knee arthroplasties recorded in the Arthroplasty Clinical Outcomes Registry National (ACORN) with complete follow-up to 6-months post-surgery. ACORN captured complications and patient-reported outcome measures for all elective THA and TKA surgeries in ten participating institutions in Australia from September 2012 to September 2018, with follow-up to April 2019. All adults undergoing elective primary or revision THA or TKA at one of ten participating sites and with the cognitive capacity to respond to patient-reported outcome questions were eligible for inclusion in the registry. In this study, patients were excluded if they had undergone revision surgery or unicompartmental knee arthroplasty, opted out of registry follow-up or were lost to follow up. Ethical approval for this study was granted by the Hunter New England Human Research Ethics Committee (HREC; reference no. 12/11/21/5.02). The HREC also approved the opt-out consent process, meaning that all eligible people were included in the ACORN Registry unless they specifically contacted the registry to opt out.

### Data collection in ACORN

Pre-operatively, ACORN participants provide demographic and anthropometric information and medical history, and complete patient-reported index joint (Oxford Knee or Hip Score [20]) and generic health-related quality of life (EuroQol 5 L-5D [21]) questionnaires. ACORN hospitals collect information about the acute stay on an ACORN proforma including complications, length of stay and discharge destination (e.g. usual residence, inpatient rehabilitation, nursing home, death). Dedicated registry staff collect six-month Oxford Knee or Hip Score and EuroQol 5 L-5D by telephone, as well as information on complications including readmission and reoperation. ACORN Registry staff entered baseline, acute-care and follow-up data into the REDCap database hosted at the University of New South Wales, Australia. ACORN data have high levels of completeness (> 99%) and accuracy (94–96%) [22].

### Outcomes

Complications were collected at two time points: during the acute inpatient stay, and at six months post-operatively. Complications captured at both time points were combined to identify people who had ‘ever’ or ‘never’ experienced the complication. We categorised complications into ‘major’, defined as those requiring complex medical intervention, and ‘minor’ (all other complications).

Major complications captured at both time points included death, reoperation, dislocation, fracture, deep vein thrombosis (DVT), pulmonary embolism (PE), surgical site infections (SSI) requiring oral antibiotics, SSI requiring intravenous antibiotics, cardiovascular complications and stroke. Arthroplasty-related readmission was captured at 6-month follow-up for which possible responses were DVT, PE, manipulation under anaesthetic, dislocation, SSI, wound dehiscence, index joint revision and other.

Minor complications captured at both time points include bladder infection or urinary retention, respiratory infection, cellulitis, joint stiffness and neuropathy. Delirium, wound dehiscence, fall during stay and hypotension were captured only during the acute admission whilst joint stiffness, unexpected pain, leg length discrepancy, swelling and persistent paraesthesia were captured only at 6 months.

### Statistical analysis

After testing for distributional assumptions, we presented patient characteristics by joint or by complication status using means or medians as appropriate for continuous variables, and number and percent for categorical variables. We estimated the association between pre-operative patient factors and outcomes using unconditional logistic regression models. All models were adjusted for age, sex, body mass index (BMI), heart disease, hypertension, diabetes, gastric, lung, renal and neurological disease, anxiety/depression, low back pain, lower limb arthritis other than operated joint, and prior hip or knee arthroplasty [23]. All analyses were conducted using Stata/SE Version 15.1.

## Results

### Characteristics of the study cohort

**Fig. 1:**
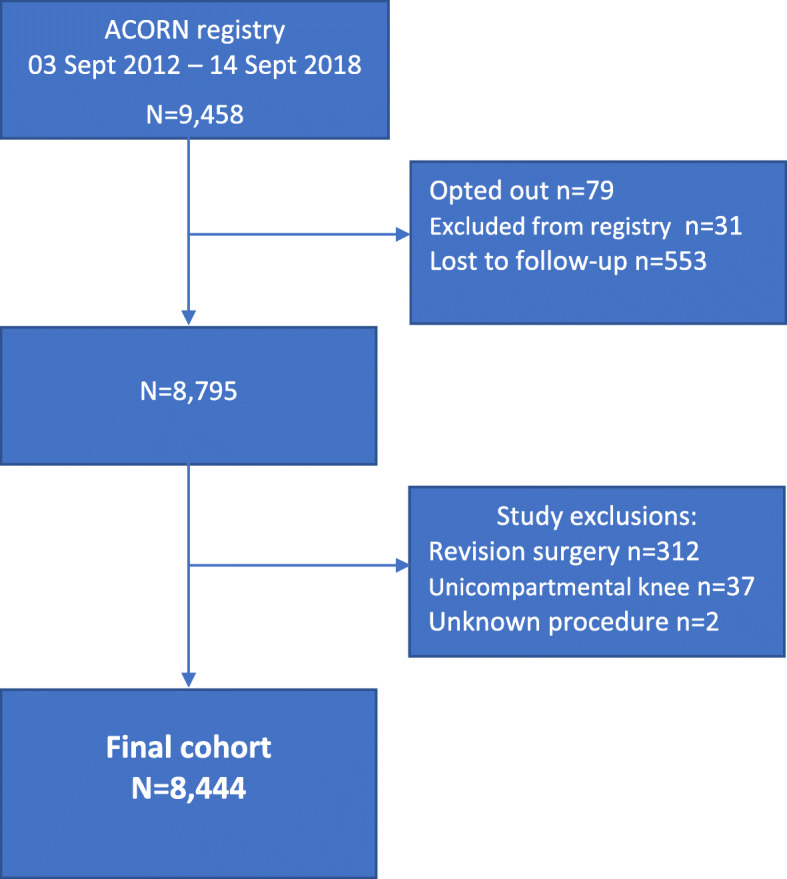
Study cohort

Of 9458 procedures captured in ACORN between September 2012 and September 2018, 8444 procedures met the inclusion criteria for this study (2782 THA and 5662 TKA). A total of 1014 procedures were excluded due to: revision surgery (*n* = 312); unicompartmental knee arthroplasty (*n* = 37); procedure data missing (*n* = 2); patient opted out (*n* = 79); patient did not meet inclusion criteria for the registry i.e. lack of cognitive capacity to respond to patient-reported outcomes (*n* = 31); and loss to follow-up (*n* = 553) (Fig. 1).

The overall mean age for THA patients was 67 years (SD 11.5) and 69 (SD 9.0) for TKA patients. Fifty-five percent of the THA cohort were female, compared with 62% of the TKA cohort. The knee cohort had a greater comorbid burden compared to the hip cohort including higher BMI (knee: mean 33.2 [SD 6.7] vs hip: mean 30.4 [SD 6.2]). Patient characteristics are summarised in Table 1.

**Table 1:** Characteristics of the study cohort by joint

	Hip *N* = 2782	Knee *N* = 5662
**Demographics**		
Age, mean (SD)	67.1 (11.5)	68.7 (9)
Male	1262 (45.4)	2157 (38.1)
**Comorbidities**		
BMI, mean (SD)	30.4 (6.2)	33.2 (6.7)
Heart disease	923 (33.2)	2170 (38.3)
Hypertension	1425 (51.2)	3435 (60.7)
Diabetes	439 (15.8)	1292 (22.8)
Stomach	488 (17.5)	1230 (21.7)
Lung	416 (15)	914 (16.1)
Kidney	153 (5.5)	315 (5.6)
Liver	58 (2.1)	135 (2.4)
Neurological	147 (5.3)	274 (4.8)
Anxiety/depression	497 (17.9)	1006 (17.8)
Low back pain	1129 (40.6)	1861 (32.9)
Other lower limb arthritis	864 (31.1)	1672 (29.5)
**Prior joint replacement**		
Previous knee arthroplasty	296 (10.6)	1272 (22.5)
Previous hip arthroplasty	547 (19.7)	315 (5.6)
**Patient-reported outcomes**		
EQ-VAS (Baseline), mean (SD)	61.5 (22.9)	65.9 (20.9)
EQ-VAS (6 months), mean (SD)	77.5 (17.8)	76.1 (17.4)
OHS or OKS (Baseline), mean (SD)	15.8 (8.2)	18.6 (8.2)
OHS or OKS (6 months), mean (SD)	42.6 (7.2)	38.4 (8)

*Abbreviations*: *SD* standard deviation, *EQ-VAS* EuroQoL Visual Analog Scale, minimum score 0 (worst) maximum 100 (best), *OHS* Oxford Hip Score, minimum score 0 (worst), maximum 48 (best); OKS Oxford Knee Score, minimum score 0 (worst), maximum 48 (best)

### Complications

Following THA, 39.7% of patients had experienced at least one major (9.5%) or minor (34.0%) complication by six months (Table 2). The most reported minor complications included stiffness (8.8%) and unexpected pain (5.5%), whereas the most common major complications were arthroplasty-related readmission (3.9%), and reoperation (2.0%) (Table 3). The most common reasons for readmission were SSI (1.5%), dislocation (0.5%) and pulmonary embolism (0.2%), while the most common reasons for reoperation were SSI (0.7%), SSI requiring prosthesis removal (0.4%) and dislocation (0.3%) (Table 4). The mortality rate was 0.2%.

**Table 2:** Proportion of procedures with a complication by timing and severity of complication

	Hip *N* = 2782	Knee *N* = 5662
**During acute admission**		
Major	80 (2.9)	173 (3.1)
Minor	246 (8.8)	540 (9.5)
Overall	311 (11.2)	679 (12)
**From discharge to 6 months**		
Major	192 (6.9)	666 (11.8)
Minor	775 (27.9)	2332 (41.2)
Overall	899 (32.3)	2675 (47.2)
**Total**		
Major	263 (9.5)	815 (14.4)
Minor	947 (34.0)	2637 (46.6)
Overall	1103 (39.7)	3033 (53.6)

**Table 3:** Complications by severity and type (includes complications during acute admission and follow-up to 6 months post-surgery)

	Hip *N* = 2782	Knee *N* = 5662
**Major**	**263 (9.5)**	**815 (14.4)**
Mortality	5 (0.2)	14 (0.2)
Reoperation	55 (2.0)	144 (2.5)
Arthroplasty-related readmission	109 (3.9)	342 (6.0)
Dislocation	11 (0.4)	4 (0.1)
Fracture	31 (1.1)	21 (0.4)
DVT	21 (0.8)	102 (1.8)
PE	10 (0.4)	33 (0.6)
Surgical site infection requiring:		
Oral antibiotics	45 (1.6)	237 (4.2)
IV antibiotics	6 (0.2)	13 (0.2)
Cardiovascular	45 (1.6)	107 (1.9)
Stroke	1 (0)	1 (0)
**Minor**	947 (34.0)	2637 (46.6)
Bladder infection or retention	78 (2.8)	133 (2.3)
Respiratory infection	29 (1)	42 (0.7)
Cellulitis	13 (0.5)	50 (0.9)
Nerve injury/ neuropathy	23 (0.8)	71 (1.3)
Delirium^a^	24 (0.9)	51 (0.9)
Wound dehiscence^a^	9 (0.3)	34 (0.6)
Pressure area^a^	1 (0)	3 (0.1)
Fall during hospital stay^a^	3 (0.1)	23 (0.4)
Hypotension^a^	48 (1.7)	43 (0.8)
Drug reaction^a^	0	2 (0)
Fat embolus^a^	0	1 (0)
Other^a^	97 (3.5)	238 (4.2)
Joint stiffness^b^	244 (8.8)	1045 (18.5)
Unexpected pain^b^	153 (5.5)	555 (9.8)
Leg length discrepancy^b^	197 (7.1)	84 (1.5)
Swelling^b^	129 (4.6)	881 (15.6)
Paraesthesia^b^	149 (5.4)	885 (15.6)
Muscle weakness^b^	64 (2.3)	123 (2.2)
**Total (major and minor)**	**1103 (39.7)**	**3033 (53.6)**

^a^ Acute admission only

^b^ Post-discharge only

**Table 4:** Reason for reoperation (post-discharge only) and readmission by joint

	Hip *N* = 2782	Knee *N* = 5662
**Reoperation**	51 (1.8%)	138 (2.4%)
SSI requiring surgery	19 (0.7)	30 (0.5)
SSI requiring surgery, with prosthesis removal	10 (0.4)	8 (0.1)
Dislocation	8 (0.3)	0
Joint stiffness	0	83 (1.5)
Peri-prosthetic fracture	4 (0.1)	2 (0.04)
Implant fracture	1 (0.04)	1 (0.02)
Bleeding	2 (0.1)	0
Other/unknown	5 (0.2)	14 (0.2)
**Readmission (arthroplasty-related)**	109 (3.9%)	342 (6.0%)
DVT	4 (0.1)	23 (0.4)
PE	5 (0.2)	8 (0.1)
Manipulation under anaesthetic	0	106 (1.9)
Dislocation	14 (0.5)	0
SSI	43 (1.5)	117 (2.1)
Wound dehiscence	1 (0.04)	4 (0.1)
Index joint revision	4 (0.1)	1 (0.02)
Other^a^	35 (1.3)	82 (1.4)

^a^The most common responses for ‘other’ reason for arthroplasty-related readmission for both joints were ‘cellulitis’ and ‘pain’; ‘swelling’ was common for knees only

Following TKA, 53.6% of people experienced at least one complication (major: 14.4%; minor, 46.6%; Table 2). The most common minor complications were joint stiffness (18.5%), swelling (15.6%) and paraesthesia (15.6%), while the most common major complications were arthroplasty-related readmission (6.0%) and reoperation (2.5%; Table 3). The most common reasons for arthroplasty-related readmission following TKA were SSI (2.1%), manipulation under anaesthesia (MUA) (1.9%) and DVT (0.4%), while the most common reasons for reoperation following TKA were joint stiffness (1.5%) and SSI requiring surgery (0.5%) (Table 4). The mortality rate was 0.2%.

Relative to THA patients, TKA patients reported higher rates of both major (knee: 14.4% vs hip: 9.5%) and minor (knee: 46.6% vs hip: 34.0%) complications, including higher rates of reoperation, readmission, DVT, SSI requiring oral antibiotics, nerve injury, stiffness, unexpected pain, swelling and paraesthesia, whilst THA patients experienced higher rates of dislocation, fracture, leg length discrepancy and bladder infection (Table 3).

### Associations between patient factors and complications

Among THA recipients, comparing people who experienced complications to those who did not, each 1 kg/m^2^ increase in BMI, measured at baseline, was associated with increased odds of reoperation (adjusted odds ratio [aOR]: 1.05; 95% CI: 1.01–1.09), arthroplasty-related readmission (aOR: 1.04; 95% CI: 1.01–1.07) and SSI (aOR: 1.07; 95% CI: 1.02–1.11) at six months. Having undergone a previous THA was associated with increased odds of reoperation (aOR: 2.35; 95% CI: 1.31–4.23) and arthroplasty-related readmission (aOR: 2.05; 95% CI: 1.35–3.14), whilst bilateral surgery was associated with higher odds of SSI (aOR: 4.81; 95% CI:1.36–17.0) (Table 5).

**Table 5:** Adjusted odds ratios for reoperation, readmission and surgical site infection at 6 months following total HIP arthroplasty

	Reoperation	Readmission (arthroplasty-related)	Surgical site infection
Patient factors	Adjusted OR^a^ (95% CI)	Adjusted OR^a^ (95% CI)	Adjusted OR^a^ (95% CI)
**Demographics**			
Age, mean (SD)	0.98 (0.96–1.01)	1.00 (0.98–1.02)	0.99 (0.96–1.02)
Female	1.19 (0.67–2.13)	0.96 (0.64–1.43)	1.53 (0.81–2.88)
**Comorbidities**			
BMI (kg/m^2^)	**1.05 (1.01–1.09)**	**1.04 (1.01–1.07)**	**1.07 (1.02–1.11)**
Heart disease	1.48 (0.81–2.71)	1.42 (0.93–2.18)	1.33 (0.68–2.60)
Hypertension	1.57 (0.84–2.94)	1.38 (0.89–2.15)	0.65 (0.34–1.25)
Diabetes	0.58 (0.25–1.35)	0.78 (0.45–1.35)	0.84 (0.36–1.99)
Stomach	1.02 (0.51–2.06)	0.93 (0.55–1.56)	0.51 (0.20–1.34)
Lung	1.36 (0.68–2.69)	1.26 (0.76–2.09)	1.97 (0.98–3.94)
Kidney	0.64 (0.15–2.70)	1.01 (0.45–2.26)	0.44 (0.06–3.29)
Liver	1.36 (0.30–6.18)	0.80 (0.19–3.48)	1
Neurological	1.18 (0.41–3.42)	0.73 (0.29–1.84)	1.14 (0.34–3.84)
Anxiety/depression	1.36 (0.70–2.64)	1.06 (0.63–1.78)	0.99 (0.46–2.15)
Low back pain	1.58 (0.86–2.88)	1.51 (0.99–2.31)	1.39 (0.73–2.64)
Other lower limb arthritis	0.88 (0.47–1.65)	0.97 (0.62–11.51)	0.74 (0.37–1.49)
**Prior joint replacement**			
Previous knee arthroplasty	0.56 (0.19–1.61)	1.12 (0.62–2.02)	1.94 (0.88–4.31)
Previous hip arthroplasty	**2.35 (1.31–4.23)**	**2.05 (1.35–3.14)**	1.38 (0.68–2.80)
**Surgical factors**			
Bilateral surgery	1.60 (0.21–12.26)	0.81 (0.11–6.03)	**4.81 (1.36–17.0)**

^a^Adjusted for all covariables shown in table

For TKA patients, age was inversely associated with reoperation (aOR: 0.96; 95% CI: 0.94 to 0.98) and surgical site infection (aOR: 0.98; 95% CI: 0.97 to 1.00), and female sex was inversely associated with arthroplasty-related readmission (aOR: 0.75; 95% CI: 0.60 to 0.95). Among TKA patients, previous hip arthroplasty was associated with 61% higher odds of arthroplasty-related readmission (aOR: 1.61; 95% CI: 1.07 to 2.43) (Table 6). Factors among TKA patients that were associated with an increased odds of minor complications were heart disease (aOR: 1.38; 95%CI: 1.23–1.55), low back pain (aOR: 1.34; 95% CI 1.19–1.52) and other lower limb arthritis (aOR: 1.28; 95% CI 1.13–1.45), whilst previous TKA (aOR: 0.79; 95% CI: 0.69–0.90) and liver disease (aOR: 0.53; 95%CI: 0.28–0.99) were associated with a decreased odds of minor complications at 6 months (aOR: 0.79; 95% CI: 0.69–0.90).

**Table 6:** Adjusted odds ratios for reoperation, readmission and surgical site infection at 6 months following total KNEE arthroplasty

	Reoperation	Readmission (arthroplasty-related)	Surgical site infection
Patient factors	Adjusted OR^a^ (95% CI)	Adjusted OR^a^ (95% CI)	Adjusted OR^a^ (95% CI)
**Demographics**			
Age, mean (SD)	**0.96 (0.94–0.98)**	0.99 (0.98–1.01)	**0.98 (0.97–1.00)**
Female	0.76 (0.53–1.07)	**0.75 (0.60–0.95)**	1.27 (0.96–1.69)
**Comorbidities**			
BMI (kg/m^2^)	0.98 (0.95–1.01)	1.01 (0.99–1.03)	1.01 (0.99–1.03)
Heart disease	0.91 (0.62–1.32)	0.94 (0.74–1.20)	0.88 (0.67–1.17)
Hypertension	0.75 (0.52–1.07)	0.80 (0.63–1.02)	1.03 (0.78–1.37)
Diabetes	0.96 (0.62–1.49)	0.92 (0.70–1.23)	1.02 (0.75–1.39)
Stomach	0.85 (0.54–1.33)	0.97 (0.73–1.28)	1.21 (0.90–1.64)
Lung	0.87 (0.53–1.44)	0.95 (0.70–1.30)	**1.43 (1.04–1.96)**
Kidney	1.84 (0.99–3.41)	1.12 (0.70–1.79)	0.87 (0.48–1.59)
Liver	0.98 (0.35–2.72)	1.56 (0.86–2.82)	0.88 (0.38–2.03)
Neurological	1.80 (0.95–3.42)	1.43 (0.91–2.26)	0.93 (0.51–1.69)
Anxiety/depression	0.92 (0.58–1.46)	1.07 (0.80–1.43)	1.15 (0.83–1.59)
Low back pain	1.19 (0.82–1.74)	1.22 (0.96–1.56)	1.28 (0.96–1.70)
Other lower limb arthritis	1.33 (0.92–1.93)	1.22 (0.95–1.56)	0.78 (0.58–1.06)
**Prior joint replacement**			
Previous knee arthroplasty	0.73 (0.45–1.16)	0.84 (0.63–1.12)	0.88 (0.64–1.21)
Previous hip arthroplasty	1.27 (0.63–2.54)	**1.61 (1.07–2.43)**	0.64 (0.32–1.26)
**Surgical factors**			
Bilateral surgery	0.61 (0.28–1.32)	0.74 (0.45–1.21)	0.51 (0.26–1.01)

^a^Adjusted for all covariables shown in table

Female sex was associated with increased odds of minor complications following THA (aOR: 1.34; 95% CI: 1.13–1.58) but decreased odds following TKA (aOR: 0.85; 95% CI 0.76–0.95). Results of adjusted regression models outlining associations between patient factors and overall major or minor complications are presented in Table 7.

**Table 7:** Adjusted odds ratios for major and minor complications to 6 months, by joint

	Major		Minor	
	Hip	Knee	Hip	Knee
Patient factors	Adjusted OR^a^ (95% CI)	Adjusted OR^a^ (95% CI)	Adjusted OR^a^ (95% CI)	Adjusted OR^a^ (95% CI)
**Demographics**				
Age, mean (SD)	1.01 (0.99–1.02)	1.00 (0.99–1.01)	1.00 (0.99–1.01)	**0.99 (0.98–1.00)**
Female	1.23 (0.94–1.61)	0.93 (0.79–1.09)	**1.34 (1.13–1.58)**	**0.85 (0.76–0.95)**
**Comorbidities**				
BMI (kg/m^2^)	**1.03 (1.01–1.05)**	1.01 (1.00–1.02)	1.00 (0.99–1.01)	1.00 (0.99–1.06)
Heart disease	1.21 (0.90–1.61)	0.95 (0.81–1.12)	1.17 (0.98–1.41)	**1.38 (1.23–1.55)**
Hypertension	0.98 (0.74–1.30)	0.93 (0.79–1.09)	0.95 (0.80–1.14)	0.94 (0.84–1.06)
Diabetes	1.10 (0.78–1.56)	1.04 (0.86–1.25)	1.16 (0.92–1.45)	0.93 (0.81–1.06)
Stomach	0.74 (0.51–1.07)	1.11 (0.92–1.33)	0.96 (0.77–1.19)	1.01 (0.88–1.15)
Lung	1.32 (0.94–1.85)	1.20 (0.98–1.46)	1.23 (0.98–1.53)	1.08 (0.94–1.26)
Kidney	1.03 (0.60–1.79)	1.12 (0.82–1.53)	1.07 (0.75–1.52)	1.08 (0.86–1.39)
Liver	0.90 (0.34–2.33)	1.07 (0.67–1.71)	**0.53 (0.28–0.99)**	0.98 (0.69–1.39)
Neurological	0.93 (0.52–1.66)	1.13 (0.80–1.58)	1.14 (0.80–1.62)	1.28 (1.00–1.64)
Anxiety/depression	1.05 (0.74–1.48)	1.06 (0.87–1.29)	1.09 (0.88–1.35)	1.13 (0.98–1.31)
Low back pain	**1.36 (1.02–1.80)**	**1.34 (1.14–1.59)**	1.16 (0.97–1.38)	**1.34 (1.19–1.52)**
Other lower limb arthritis	0.80 (0.59–1.08)	1.02 (0.86–1.21)	**1.29 (1.08–1.55)**	**1.28 (1.13–1.45)**
**Prior joint replacement**				
Previous knee arthroplasty	1.14 (0.77–1.70)	0.83 (0.69–1.00)	1.23 (0.95–1.60)	**0.79 (0.69–0.90)**
Previous hip arthroplasty	1.29 (0.95–1.75)	1.18 (0.87–1.62)	0.98 (0.80–1.20)	0.84 (0.66–1.06)
**Surgical factors**				
Bilateral surgery	0.84 (0.25–2.77)	0.72 (0.51–1.02)	1.69 (0.90–3.16)	1.15 (0.92–1.44)

^a^Adjusted for all covariables shown in table

## Discussion

### Summary of main findings

In this analysis of data from an arthroplasty registry in Australia, we found high overall rates of post-operative complications, with 9.5% of THA and 14.4% of TKA patients having experienced a major complication by 6 months post-surgery; 34.0% of THA and 46.6% of TKA patients experienced a minor complication over the same time frame.

### Comparison with previous studies

#### Major complications

Complication rates in our registry were higher than those reported by NSQIP, which reported major and minor 30-day complication rates at 4.2 and 2.17% for THA and 1.83 and 3.20% for TKA, respectively [6, 18]. However, our registry follows patients to 6 months rather than 30 days; this longer follow-up period may account for at least part of the difference in complication rates. Our time period was chosen to allow adequate time for improvement in function and complications to surface [24].

#### Infections

We found higher rates of surgical site infections to those in the literature. In our TKA cohort, for example, 4.2% and 0.2 of patients reported SSI requiring oral or IV antibiotics, respectively. A Hong Kong study analysing elective unilateral TKA by a single surgeon over 10 years observed rates of SSI requiring oral antibiotics at 0.66%, whilst a slightly larger Taiwanese study reported a rate of 1.52% [25, 26]. A large UK prospective study showed superficial SSI at 2.23% for THA patients compared to our rates of 1.6 and 0.5% requiring oral and IV antibiotics, respectively [27]. Surgical site infections are important as they can lead to prosthetic joint infections (PJI) which require revision surgery [28]. Rates of PJI requiring revision surgery have been observed in large international studies including the US, Europe, Australia and New Zealand at 0.6–1.6% for THA and 0.7–1.5% for TKA [12, 29–33] which is higher than the rates found in this study (THA = 0.4%, TKA = 0.1%). However, this is unsurprising as most studies investigating PJI observed a period of 12- to 24-months post-operatively compared to the 6-month period in this study. Our high rates of oral antibiotic use may reflect low prescribing thresholds rather than an underlying difference in superficial infections.

#### VTE

We found DVT rates of 0.8 and 1.8% for THA and TKA. These are higher than reported in previous studies using inpatient data in the US (0.24 and 0.45% for THA and TKA, respectively) and China (0.24% vs 0.71%), but lower than 90-days post-operatively in Korea (2.4% vs 3.4%) %) [15, 34, 35], whilst a meta-analysis of patients receiving chemoprophylaxis following THA and TKA published rates of VTE at 1% for TKA and 0.5% for THA, again limited to the inpatient stay [16]. The variability seen in these rates may be due to both patient factors including comorbidities and cultural factors, and clinical factors, as such as mechanical and chemoprophylaxis. Previous studies have found higher rates of DVT in patients undergoing TKA compared to THA, and this was reflected in our study with higher rates of both PE and DVT in TKA patients compared to THA. This may be explained by the involvement of smaller calf veins that are affected in TKA compared to larger veins in THA which would take longer to become occluded and therefore symptomatic [16]. Tourniquet use may also contribute to the higher rate of DVT in TKA [36].

#### Mortality and readmission

At 6 months, all-cause mortality rates were 0.2% for both procedures, which falls within the range of published 30- and 90-day rates of 0.05–1.1% in the US, UK, Australia and Denmark [37–42]. Compared to readmission rates from US Medicare data, we found lower readmission rates at 6 months than the 30-day rates for THA and 90-day rates for TKA [8, 9, 43]. We found that infection was overall the most common reason for arthroplasty-related readmission in both procedures, followed by periprosthetic fracture and dislocation for THA and cellulitis for TKA. A single centre study at a US orthopaedic specialty hospital reported significantly lower rates of 0.33 and 0.21% for THA and TKA, respectively, at 30 days [17]. However, those numbers are limited to patients who re-present to the same hospital, and therefore those who present to different centres may not have been captured using this metric. Our data is advantageous in this regard as data are collected directly from patients and do not rely on re-presentation to the same institution.

#### Minor complications overall

We found significantly higher rates of minor complications in this study at 34.0% for THA and 46.6% for TKA compared to published rates of 2.7% for THA and 3.2% for TKA from NSQIP data [6, 18]. The NSQIP data use hospital medical charts documented by medical staff to capture complications, whereas our registry used a combination of hospital data for the acute stay along with patient-reports collected by telephone interview at 6 months. In addition to ACORN registering a larger range of patient-reported minor complications, including hypotension, swelling and paraesthesia, it is possible that patients and surgeons have different conceptions of what constitutes a complication. For example, stiffness may indicate patients’ subjective experience, whilst rates of MUA may suggest an objective measurement by surgeons that indicates stiffness severe enough to require intervention. Disagreement in complication reporting rates between clinicians and patients has already been established following orthopaedic and general surgery [44–48]. Although minor complications such as stiffness, swelling and paraesthesia do not necessarily indicate procedure failure, they represent legitimate patient experience, and the absence of measurement standards to characterise their severity may impact the accuracy and reliability of complications data. Further, minor complications may reflect quality of care delivery. For example, high rates of pressure sores may reflect a lack of attention to early ambulation, while high rates of hypotension may reflect too high a dose of intra-operative opioid. Even minor complications are costly to the health system and may be important to patients thus are worth preventing.

#### Patient characteristics associated with complications

Our study found low back pain to be an independent predictor for major complications following either THA or TKA, and BMI as a risk factor for major complications following THA. BMI, longer operative time and higher American Society of Anaesthesiologist scores have been identified as independent predictors of postoperative complications overall in both THA and TKA patients [13, 49]. Bleeding disorder and anaemia have further been identified as risk factors for major complications following THA, and low back pain has been shown to influence functional outcomes following arthroplasty procedures [6, 18, 50, 51]. Further, in separate risk-adjusted models we found increasing age to be associated with reduced odds of reoperation, and female gender to be associated with decreased odds of readmission. These associations may reflect the selection of patient groups who have lower comorbidity load as eligible and appropriate for surgery. Geriatric patients aged over 85 are significantly less likely to receive TKA than their younger counterparts; it may be that among patients who do undergo arthroplasty, increased age reflects the selection process of appropriate surgical candidates rather than being a protective factor, as only patients with limited comorbidities who are likely to experience long term benefits would be considered for total arthroplasty procedures [6]. Increased BMI in THA was the single patient factor that was consistently positively associated with major complications, including infection, reoperation and readmission which is in accordance with previous studies which showed BMI to have strong associations with dislocation, infection and revision [52, 53]. A study using the New Zealand Joint Registry also reported an OR of 3.73 for PJI in patients with BMI > 40 compared to patients with BMI < 35 [54].

Increased age, female gender and previous knee arthroplasty were associated with decreased odds of experiencing a minor complication for TKA patients. Although male gender has been identified in the literature as an independent predictor for postoperative complications due to higher rates of comorbidities such as diabetes, hypertension and smoking, we found increased odds of minor complications among women undergoing THA [55].

### Strengths and limitations

A strength of this study is that it is a large cohort with high rates of long-term follow up. Further, contacting patients directly means that complications managed in the community or a different healthcare facility would have been captured which is a potential limitation of studies relying on single centre administrative data [16]. Limitations of our study include potential recall bias, accuracy of patient reports and although not necessarily a drawback of this study, we were unable to make fair comparisons with other similar studies due to differences in follow up time and complication definitions. We did not capture the timing of complications that occurred after hospital discharge, so were not able to determine whether they happened shortly after discharge or later in the 6-month follow-up period. Although complications such as bladder infection or retention and respiratory infection may be expected during the acute hospital admission, after discharge, particularly after the first 30 days, they are less likely to be arthroplasty-related. Depending on timing, it is possible that some of these complications were not related to surgery and thus may overestimate their true incidence in relation to the procedure. ACORN also did not capture care characteristics, for example, type of VTE or infection prophylaxis received, thus we are unable to provide complication outcomes according to care received.

## Conclusions

This is the first study that uses Australian multicentre data to provide an overview of post-operative complication rates following THA and TKA. We found moderate rates of major complications and high rates of minor post-operative complications, and found several patient factors such as female sex and age to be protective for certain complications in TKA patients, whilst increased BMI and bilateral surgery in THA patients were risk factors for complications. Efforts should be focused on further identifying patients with higher risk and optimising pre- and post-operative care to reduce rates of these complications.

## Data Availability

The data is available at Research Data Australia at: https://researchdata.edu.au/acorn-arthroplasty-clinical-registry-national/1461683/?refer_q=rows=15/sort=score%20desc/class=collection/p=1/q=acorn/

## Abbreviations

TKA: Total Knee Arthroplasty
THA: Total Hip Arthroplasty
NSQIP: National Surgery Quality Improvement Program
ACORN: Arthroplasty Clinical Outcomes Registry National
DVT: Deep vein thrombosis
PE: Pulmonary embolism
SSI: Surgical site infection
BMI: Body Mass Index
MUA: Manipulation under anaesthesia
PJI: Prosthetic joint infection
VTE: Venous thromboembolism
